# Beginning: China's national park system

**DOI:** 10.1093/nsr/nwac150

**Published:** 2022-08-02

**Authors:** Weijie Zhao

**Affiliations:** NSR news editor based, Beijing

## Abstract

At the leaders’ summit of the 15th Meeting of the Conference of the Parties to the Convention on Biological Diversity (COP15) on 12 October 2021, China's President Xi Jinping declared that China has officially designated its first group of national parks—the Three-River-Source National Park, the Giant Panda National Park, the Northeast China Tiger and Leopard National Park, the Hainan Tropical Forests National Park, and the Wuyishan National Park. The five national parks cover a total area of ∼230 000 square kilometers and protect nearly 30% of the key terrestrial wildlife species found in China.

Since the establishment of the United States’ Yellowstone National Park in 1872, national parks have been founded in many countries, and some of them have become distinctive ‘national signatures’ and important ecological security shelters. As a large country with rich biodiversity, China now begins to establish its first five national parks, with a total of about 50 planned for the future, and to form a national-park-centric protected-area system. How will national parks change the landscape of China's ecological conservation? Is China well prepared to scientifically establish and administrate national parks? In this panel discussion, six Chinese ecological conservationists introduce the background, plans and challenges of China's national park system, and provide their scientific perspectives.

Guangchun Lei

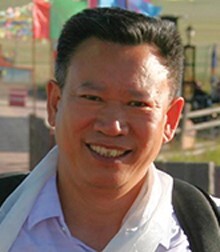

Professor, School of Ecology and Nature Conservation, Beijing Forestry University

Zhiyun Ouyang

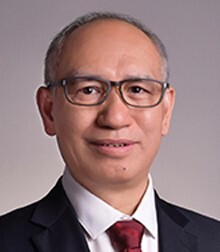

Professor, Research Center for Eco-Environmental Sciences, Chinese Academy of Sciences

Yang Su

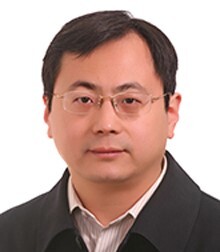

Research Fellow, Development Research Center of the State Council of China

Rui Yang

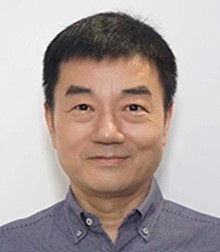

Professor, Institute for National Parks, Tsinghua University

Yujun Zhang

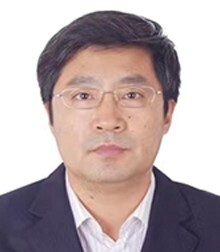

Professor, School of Landscape Architecture, Beijing Forestry University

Keping Ma (Chair)

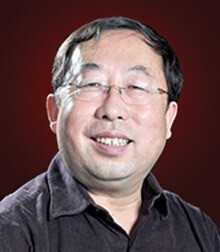

Professor, Institute of Botany, Chinese Academy of Sciences

## Which kind of ‘park’?

**KP Ma:** Welcome to today's panel discussion. Our first question is: what is a national park?

**R Yang:** It is easy for the public to consider national parks as similar to the ‘parks’ they know: the parks for relaxation and enjoyment. But actually, that is only a small part of the function of China’s national parks. Basically, a national park is a type of natural protected area. National parks fundamentally aim to protect ecosystems, especially large-scale and dynamic ecological processes, and also provide chances for scientific, educational and recreational purposes. A national park is not a tourist site, nor a strict nature reserve forbidding public access.

Why do societies establish national parks? The relationship between humans and nature has evolved with human civilization: in the hunting and gathering era, humans were a part of nature; after agricultural civilization began, humans acquired their own lands, but still kept a balance with the rest of nature; since

A national park is not a tourist site, nor a strict nature reserve forbidding public access.

——Rui Yang

the Industrial Revolution, humans have become the ‘ruler’ of the planet—we occupy >80% of the Earth's land. Humans have a responsibility to protect nature. The world's first national park, the Yellowstone National Park, was established with such a background. The western development of the US brought irreversible destruction to the western wilderness and the American Indian civilization, so George Catlin suggested establishing a national park to protect the natural and cultural heritage. So, the national park is a reflection of the change in the human–nature relationship, and is becoming more and more important under the crises of climate change and biodiversity loss.

Ancient Chinese philosopher Chuang Tzu wrote: ‘The mountain trees invite their own cutting down; lamp oil invites its own burning up. Cinnamon bark can be eaten; therefore the tree is cut down. Lacquer can be used, therefore the tree is scraped. All men know the utility of useful things; but they do not know the utility of futility.’ These passages are an important exposition of the human–nature relationship. We cut down trees for their ‘utility’, but the towering trees that cannot be readily used and seem to be ‘futile’ are actually the more important ‘great utility’. I think national parks should play a significant role in realizing the utility of ‘futility’, and in helping to achieve harmony between humans and nature. Nature is not just resources for humans, rather it is the shared habitat for humans and all species.

**KP Ma:** What are the unique characteristics of China's national parks?

**R Yang:** China's national parks are quite unique. First, they are emerging at a special time when human civilization is transiting from industrial to ecological civilization. The Chinese government proposed the concept of ecological civilization in 2007, decided to construct the national park system in 2013, and proposed the national-park-centric protected-area system in 2017. Against the background of ecological civilization, we will build our national parks in a more scientific and systematic way.

Second, the national park system in China will be the largest in the world. The average land area of the first five national parks is 46 000 square kilometers, with the largest, the Three-River-Source National Park, covering nearly 200 000 square kilometers. As a comparison, the average area of the 62 US national parks is <3400 square kilometers. China may build 50 to 80 national parks, covering about 10% of the country's entire land area, whereas this ratio is 2.3% in the US, and ∼3.4% worldwide.

Third, the speed of establishing national parks is very fast. It took only eight years from the proposal of the construction plan in 2013 to the formal establishment of the first national parks in 2021. In particular, it took only three years from the proposal to the establishment of the Hainan Tropical Forests National Park.

Fourth, the central government has made a firm decision to implement strong protection measures. In the original proposal, experts used the wording ‘ecological protection has a high priority’ and ‘more strict protection’, but the government changed them to ‘ecological protection first’ and ‘the most strict protection’ in the formal document. President Xi called the construction of national parks one of the ‘top priorities of the country’ multiple times, which fully reflects the determination of the central government with regard to ecological protection.

Moreover, the construction process will be difficult. Many areas designated to national parks are underdeveloped areas with a lot of local communities, thus there will be many challenges, including complex land ownership and community livelihood, as well as the balance of food security and ecological security. The construction of national parks in China will encounter many more difficult problems than those in other countries.

**KP Ma:** How were the first national parks selected? What are the layouts? And what are the plans for the future?

**ZY Ouyang:** China's national parks will be constructed to protect national ecological security, to preserve valuable natural resources for our future generations and to provide the basis for China's sustainable development. To achieve these goals, there are four major considerations for the layout of national parks.

First, national parks should be representational for the country—they should represent the unique ecosystems in eco-geographic regions, unique landscapes and the unique species of China. Actually, these representational factors are often found together. For example, giant pandas live in the Qinling Mountains, gibbons live in the tropical forests of Hainan—these areas host both representative species and ecosystems.

Second, national parks should be of great significance to national ecological security. For example, the three-river-source area is the water tower of Asia and China; it provides 40% of the water of the Yellow River Basin and is extremely important for the ecological security of China. The tropical forests in Hainan have a great capacity for water conservation, and are the major water source of Hainan Island during the dry seasons.

Third, these areas should have relatively high authenticity and integrity. Authenticity means that these areas have not been significantly disturbed by human activities, and are well-preserved in their natural conditions. Integrity means that the ecosystem structures and eco-processes are preserved naturally, and the protected area is big enough to support the ecosystem processes and the proliferation of their representative species. We need to carefully investigate and design the demarcation of the parks.

The most important thing is that we should protect all the areas that deserve protection, and establish an integral national park system in China.

—Zhiyun Ouyang

Fourth, areas that are already well-protected and well-managed are preferred, because it is easier for these areas to be transformed into a part of the national park system.

With these considerations, we selected potential national parks step by step. First, with reference to the studies on eco-geography and vegetation regionalizations of China, we selected >200 candidates from ∼3800 natural sites that are worth protection throughout China. After that, experts from multiple fields were invited to evaluate the authenticity and integrity of these areas, and identified >80 areas suitable for national park construction. Among these areas, five important and well-managed ones were chosen as the first batch of national parks.

By 2035, there will be about 50 national parks in China, and eventually the number may reach 80. The final number may vary, but the most important thing is that we should protect all the areas that deserve protection, and establish an integral national park system in China.

**GC Lei:** I have two additional suggestions for the spatial layout of national parks. Firstly, we should consider the evolution of ecosystems. The global climate is changing and that will influence species migration and the spatial distribution of biodiversity. Therefore, we should protect the eco-areas that will be important in the future, but may appear unimportant at present.

Secondly, we should consider regional balance. Besides protection, national parks also provide the public with the opportunity to experience and learn about nature. Hence, it is important to ensure access to national parks for all citizens—the national parks should not be too far away from where people live.

## The protected-area system of China

**KP Ma:** China has started to construct a national-park-centric protected-area system. What will this system be like? Why should it be centered around national parks?

**GC Lei:** The protected-area system will be constructed of not only national parks, but also other types of protected areas such as natural reserves and natural parks. In this system, national parks will be the mainstay, natural reserves will be the basis, and the natural parks will be a supplement.

There are two reasons to center the system around national parks. First, national parks will occupy the largest total area of all types of protected areas. Second, as large-scale protected areas, national parks will provide the most systematic protection to complex and dynamic ecological processes, and to the authenticity and integrity of ecosystems.

**ZY Ouyang:** According to the current plan, we will build the most important and most representational areas into national parks, thus these national parks will definitely be the most significant component of the protected-area system.

**KP Ma:** The protected-area system we are talking about focuses upon the *in situ* conservation of biodiversity, while *ex situ* conservation is also an important conservation strategy. The China

Maybe in the future, we can build a ‘national *ex situ* conservation system’, and combine it with the protected-area system to form a complete ‘national biodiversity conservation system’.

—Keping Ma

National Botanical Garden, formally established on 18 April 2022, is an example of the *ex situ* conservation of plants. I am considering that, maybe in the future, we can build a ‘national *ex situ* conservation system’ and combine it with the protected-area system to form a complete ‘national biodiversity conservation system’.

Now, in China, the *ex situ* conservation of plants is more systematic than that of animals. We have large-scale botanical gardens, germplasm banks and germplasm gardens, as well as many herbaria and laboratories. These infrastructures are able to support *ex situ* conservation and scientific study of various precious and endangered plants. There are also successful cases of *ex situ* animal conservation in and out of China. For example, through captive breeding and associated reintroduction, the population of crested ibis rose from 7 in 1981 to >5000 now, and rescue and breeding stations played significant roles in the conservation of giant panda. On the other hand, many conservationists do not consider traditional zoos, which aim to exhibit and cater for curiosity, to be an effective way to protect wild animals. And the *ex situ* conservation capability of zoos needs to be improved. Anyway, we may consider how to integrate all *ex situ* conservation measures into a nationwide system.

Furthermore, how to effectively integrate *ex situ* and *in situ* conservation to provide the best protection to all endangered species is a topic worth consideration.

**YJ Zhang:** Yes, it's important to integrate *ex situ* and *in situ* conservation. Taking plants as an example, *ex situ* conservation is essential for rescuing many endangered species. For instance, *Clematis acerifoli* and *Vitis baihuashanensis* are found only around the Baihuashan Mountain of Beijing, and are on the brink of extinction. It is necessary to collect and cultivate these plants *ex situ*.

On the other hand, the *ex situ* conservation of plants is facing some challenges. For example, we should consider the concept of isotherm when building botanical gardens. Some plants cannot grow healthily or maintain their natural ecotypes in *ex situ* environments. For instance, Chinese pines widely distribute across northern China on altitudes above several hundred meters. When planted in low-altitude cities and gardens, their growth is often not ideal. So, I think a single National Botanical Garden in Beijing is not enough for the future *ex situ* conservation system. We need multiple botanical gardens distributed across China to preserve all kinds of plants in China.

Currently, some Chinese botanical gardens, such as the Qinling Botanical Garden, are trying to combine *in situ* and *ex situ* conservation by protecting local plants and cultivating non-local plants at the same time. I think these are effective approaches.

## National parks: the most beautiful lands in Beautiful China

**KP Ma:** The 2019 ‘Guidelines for Establishing a National-Park-Centric Protected-Area System’ called national parks ‘an important symbol of Beautiful China’. What role can national parks play in the construction of Beautiful China?

**Y Su:** What is Beautiful China? If it just means blue sky, green water and clean soil, what's the difference between that and Beautiful US or Beautiful France? These common pursuits, which characterize a better living environment for humans, cannot represent the entire environment and unique beauty of a country. Only the representational resources of national parks and a protected-area system can reflect the unique beauty of China. Thus, establishing the national-park-centric protected-area system is precisely the right move to build Beautiful China. This is an important measure to take after people's living environment is improved. It is relatively easy to repair a human's living environment, but the ecosystem, biodiversity and gene resources are difficult to recover and are more critical for the sustainable development of our society and nation. That is why the central government's document identified the protected areas as ‘a precious treasure of China, an important symbol of Beautiful China, and is of the upmost importance in maintaining the nation's ecological security’.

China aims to basically realize Beautiful China in 2035, and as mentioned by Prof. Ouyang, we will have established about 50 national parks by that time. The national park system will assume its complete shape and represent the new look of Beautiful China, the new system of ecological civilization, or we may say, the ‘new form of human civilization’ that will achieve harmony between humanity and nature, and support the sustainable development of both China and the entire world.

**R Yang:** The national parks will be the most beautiful lands in Beautiful China. Moreover, Beautiful China means not only the beauty of nature, but also the beauty of the human mind. A national park is not only a protected area, but also a great place to cultivate the goodness of human nature.

**KP Ma:** That's right, and this raises another question: what should we do to make national parks a good platform for the public to enjoy nature and to develop inner goodness?

**YJ Zhang:** We should build a public-sharing system for national parks by building platforms for scientific research, natural education, ecotourism, social services and commercial projects. A small part of national parks will be open to the public, providing opportunities for people to experience nature, to learn about nature and to participate in the protection of nature. It needs the effort of both managers and scientists. According to what I have learned, many national parks and natural reserves in China are making great efforts on this front. For example, the Three-River-Source National Park is cooperating with SinoMaps Press to promote natural education by various means, including but not limited to the writing of introductive texts, the editing of nature-related books and the promotion of creative nature writing.

A key question on natural education is: What kind of knowledge should we convey to the public? Firstly, we can tell the public what national parks are and why we build them. And for each national park, we can tell its unique and vivid natural stories. For example, in the Northeast China Tiger and Leopard National Park, we can tell the story of squirrels and fallen trees: In the early years, we used to clear all the fallen trees in the forest; but later we noticed that squirrels regularly hide pine seeds in the fallen trees, and the seeds would germinate in the rotten wood and grow into new trees; after learning that, we have adjusted the way we deal with fallen trees. With such a story, the public will easily gain an understanding of natural processes, as well as scientific ways to protect nature.

For each national park, we can tell its unique and vivid natural stories.

—Yujun Zhang

## TO CONSERVE SCIENTIFICALLY

**KP Ma:** We talked about the concept, layout and value of China's national parks. Now let's move on to the details: how can we scientifically protect biodiversity and ecosystems in national parks?

The first issue is how to scientifically delimit the boundaries of national parks. To ensure the integrity of the ecosystem, we should analyze each national park individually; we cannot set a single size standard for all national parks. If we want to protect the Tibetan antelopes, we need to consider their long-distance migration, so we need to protect a large area—from Qiangtang, the Altun Shan to Hoh Xil; 400 000 square kilometers may be a reasonable area for the integrity of the ecosystem, rather than the 200 000 square kilometers currently assigned for the Three-River-Source National Park. By contrast, for the national parks in Eastern China, where evergreen broad-leaved forests are the main protection targets, relatively small areas would be enough to protect the local ecosystem.

**Y Su:** Indeed, different ecosystems and species need different protection approaches, and protection of nature is not equivalent to prohibition of human activities. For example, different animals act differently towards human-built roads. Pandas are very sensitive and tend to stay far away from roads, so we should be extremely careful in road planning in these areas to avoid the fragmentation of the panda habitat. However, many other animals, including the ungulates on the Qinghai-Tibet Plateau and many ungulates, carnivores and birds in the agroforestry ecotone areas, are actually highly adaptive to low-grade roads. Like humans, they love to walk on these flat roads—usually at different periods of the day (maybe at night), and occupy different ecological niches. Thus, in field studies, we often find that biodiversity along the roads is surprisingly high—the infrared cameras can capture many wild species. Even for the relatively high-grade roads, such as the Qinghai-Tibet Highway, it's possible to guarantee the activities of Tibetan antelopes and human traffic at the same time, as long as we can reasonably arrange the time of human traffic in the animals’ migrating seasons.

So, instead of simply forbidding all human activities in the core protection areas of national parks, we should consider the unique characteristics and requirements of the flagship species of each park, and scientifically design individualized conservation strategies for each of them.

**GC Lei:** Science-based conservation means a lot of details. In China, when we talk about strict nature conservation, it often means strict control of forest fires, prohibition of grazing and, in some cases, relocation of local residents. But these strategies are in conflict with natural laws. In nature, forest fire is a natural periodical phenomenon that is important for the dynamic balance of animal and plant populations. In Finland and many other countries, the conservation workers purposely set fires at specific times and places to regulate local ecosystems. Moreover, about the prohibition of pasture, there was an interesting case in the UK. They forbade grazing in an area to protect local butterflies. But 20 years later, the local plant community changed a lot and all the butterflies were gone.

**KP Ma:** Ecological migration is another issue of concern. I remember that when Mount Fanjing in Guizhou Province applied for the designation of World Natural Heritage site, the application document stated that many local residents were relocated from the protected area in order to better administrate and protect the ecosystem. International experts were quite confused with this, since they regard local residents as a part of the ecosystem. We can guide their behaviors but should not relocate them.

**ZY Ouyang:** A ban on grazing may change local vegetation in some cases, but in other cases, wild herbivores would take the place of cattle and sheep to graze the grass, and help to maintain a balance. That is how the population of Tibetan antelope and Tibetan wild ass increased. According to monitoring, there were ∼60 000 Tibetan antelopes living in the Three-River-Source

In China, when we talk about strict nature conservation, it often means strict control of forest fires, prohibition of grazing and, in some cases, relocation of local residents. But these strategies are in conflict with natural laws.

—Guangchun Lei

National Park, and it is estimated that the total population has recovered to ∼300 000.

**KP Ma:** I am skeptical about the number 300 000. I am not sure whether it is a result of field investigation. The real number cannot be that large.

**GC Lei:** The uncertainty of population statistics is a great problem in China. Some experts claim that there are 200 000–300 000 goitered gazelles in China, but the database of the International Union for Conservation of Nature (IUCN) suggests that there are only 42 000–49 000 such animals worldwide.

## Challenge: to build an effective administrative system

**KP Ma:** What are the major challenges in the construction of China's national parks?

**Y Su:** The key challenge is to balance the relationship between conservation and development. That is also the key challenge of national parks worldwide. The most urgent task now is to clarify the responsibilities, authorities and benefits of local governments. If we cannot handle this issue well, local governments and local residents will be pushed to the other side of the debate.

Actually, the rapid construction of China's national parks started from the 2017 Qilian Mountain Natural Reserve pollution event. This event triggered the approval of the Qilian Mountain National Park construction plan on 26 June 2017, and the approval of the overall plan for establishing a national park system in China on 27 July 2017. From then on, the central government's Ecological and Environmental Protection Supervision Committee began to strictly supervise protected areas in China. This supervision corrected many pollution problems, but unfortunately, as the supervision committee used the outdated ‘Regulations of the People's Republic of China on Natural Reserves’ as the only legal basis, they also incorrectly punished some reasonable human activities in protected areas, and brought great stress to local governments. Some local governments began to consider the construction of national parks as the setting up of restricted zones, and to believe that it harms local economic development. This has been a great impediment to the construction of the national park system.

One example is the Chongmingdongtan National Nature Reserve in Shanghai. It was well protected and managed. Its *Spartina-alterniflora*-controlling project lasted for more than a decade and had great effect. However, the supervision committee declared that its well-managed popular science center was a ‘high-grade government reception facility’ and should be considered a negative example. The local government finally clarified the misunderstanding, but as a result, the construction plan for the Yangtze River Estuary National Park was delayed.

We really need to scientifically clarify the responsibilities, authorities and benefits of local governments to solve these problems. Local governments need to know what they can and cannot do, and should not feel afraid that they may be punished for their efforts in national park construction. If we cannot solve this problem, it will be rather difficult to establish 50 national parks by 2035.

Moreover, as President Xi said, ‘lucid waters and lush mountains are invaluable assets’. Conservation and development are not irreconcilable. We should learn to utilize natural resources in new scientific and sustainable ways, not the depletive old ways. For example, the rich natural biological gene pool is providing new opportunities for modern agriculture and drug discovery, and ecotourism and ecological industrialization will bring benefits to local people. Only in this way can local governments and people join the community interested in national park construction, and promote the sound development of the national park system.

**YJ Zhang:** How to benefit local people is a big issue. As a result of the construction of the Northeast China Tiger and Leopard National Park, many local Forestry Bureau staff members, whose job was to cut trees, need to find new jobs. Some of them can become eco-protection workers; some can enter the ecotourism industry; but a large number of them have to search for completely new opportunities. How to bring profits to local people is a question that needs to be answered.

**ZY Ouyang**: It's much more difficult to build an administrative system than to design the layout of national parks. During the evaluation of the national park candidates, we found that the reorganization of the administrative bodies was a great challenge. When integrating existing protected areas and parks into national parks, administrative bodies should be integrated into one management agency. In the case of the Giant Panda National Park, which covers three different provinces, there were as many as several dozen administrative units to be integrated. It involved the issues of job transfer or unemployment of many staff members, and was very difficult.

Moreover, we have not clarified the principles of national park construction. The newly formed national parks do not know how to delimit core protection areas and general protection areas. They do not know what they should and should not do in the different areas: are resource exploitation and tourism activities allowed, and to what extent? How will local residents be guided to a new sustainable lifestyle? All these questions need to be answered according to clear laws and rules, and that is the prerequisite for the construction and development of national parks.

The most urgent task now is to clarify the responsibilities, authorities and benefits of local governments.

—Yang Su

## The institutes of national parks

**KP Ma:** A number of research institutes related to national parks have been established in the past few years in China. There are generally three types: first, institutes established by national parks themselves; second, institutes established by the administrative agencies, such as the Institute of National Parks co-established by the National Forestry and Grassland Administration and Chinese Academy of Sciences, led by Prof. Ouyang; third, institutes established by universities or other research institutions, such as the Institute of National Parks of Tsinghua University, led by Prof. Yang. What is the research focus of these institutes, and how will they contribute to the development of national parks in China?

**GC Lei:** I suggest that institutes established by the national parks can be constructed in the manner of open research platforms. They may have staff researchers focusing on their specific research topics, and may also be open for all scientists and provide them with resources and platforms for any research topic on ecology and biodiversity.

**ZY Ouyang:** We hope that the Institute of National Parks co-established by the National Forestry and Grassland Administration and Chinese Academy of Sciences can become a platform that unites experts to perform targeted and prospective research, and to formulate theories, solutions or policy suggestions for the construction and administration of national parks.

We will conduct research on both general problems of national parks, and issues specific to individual parks. The general problems include basic scientific problems, policy issues and management technologies, such as the evaluation standard for the authenticity and integrity of ecosystems, the responsibilities and authorities of different administrative departments, and the appropriate infrared camera arrangement to monitor wild animal behaviors without disrupting them. The specific requirements of individual parks can be variable, for example, which kind of tree crown is most suitable for Hainan gibbons, how to restore habitats, or how to build roads in the Giant Panda National Park. In any case, we hope to gather the requirements of all national parks and join other researchers to find solutions.

**R Yang:** As a university-organized research institute, the Tsinghua Institute for National Parks will pay more attention to questions that are basic and general, such as the general rules of harmony between nature and humanity. We are also researching ‘climate shelters’ with regard to protected areas under the circumstances of climate change, trying to provide scientific support for the long-term planning of national parks and the protected-area system.

I think it's a good thing to have many institutes of national parks. The Earth needs biodiversity, and research on national parks also needs diversity. Different types of institutes can conduct research from different angles, and all the results will be integrated to promote the development of national parks and provide a solid basis for eco-civilization.

**KP Ma:** Thank you all for your insightful views and discussions today. I believe suggestions from experts in various fields are critical for the timely adjustment of strategies for the establishment and development of China's national parks and the protected natural land system.

